# A Context Dependent Interpretation of Inconsistencies in 2D:4D Findings: The Moderating Role of Status Relevance

**DOI:** 10.3389/fnbeh.2017.00254

**Published:** 2018-01-17

**Authors:** Kobe Millet, Florian Buehler

**Affiliations:** Department of Marketing, Vrije Universiteit Amsterdam, Amsterdam, Netherlands

**Keywords:** 2D:4D, digit ratio, social status, economic decision making, performance, dominance, context

## Abstract

Whereas direct relationships between 2D:4D and dominance related attitudes or behavior often turn out to be weak, some literature suggests that the relation between 2D:4D and dominance is context-specific. That is, especially in status-challenging situations 2D:4D may be related to dominant behavior and its correlates. Based on this perspective, we interpret inconsistencies in the literature on the relation between 2D:4D and risk taking, aggression and dominance related outcomes and investigate in our empirical study how attitudes in low 2D:4D men may change as a function of the status relevance of the context. We provide evidence for the idea that status relevance of the particular situation at hand influences the attitude towards performance-enhancing means for low 2D:4D men, but not for high 2D:4D men. We argue that 2D:4D may be related to any behavior that is functional to attain status in a specific context. Implications for (economic) decision making are discussed.

## Introduction

“Apart from economic payoffs, social status (social rank) seems to be the most important incentive and motivating force of social behavior.”

Harsanyi (1976), p. 204.

In the present article, we focus on the potential importance of the drive for social status when studying relationships between 2D:4D and risk taking, performance, overconfidence, aggression or any other behavior that may be functional to attain status. We will argue that John Harsanyi’s proposition of social status as one of the most important drivers of social decision making may especially hold for low 2D:4D men. Accordingly, we aim to illustrate how our status striving perspective may shed light on some puzzling inconsistencies in previous findings and provide some empirical evidence in support of our reasoning. Finally, we will discuss how these insights may be of relevance for the study of 2D:4D as a biological driver of economic decisions people make.

The second to fourth digit ratio or shortly 2D:4D is a biological marker referring to the relative length of the index (2nd digit) to the ring (4th digit) finger of someone’s hand. A lower 2D:4D is assumed to be the result of prenatal exposure to increased levels of testosterone (Manning, 2002) and some direct evidence is provided in non-human mammals, for instance it has been shown that the enhancement of prenatal testosterone reduces 2D:4D in rats (Talarovicová et al., 2009) as well as in mice (Zheng and Cohn, 2011). Moreover, a lot of indirect evidence in humans speaks towards this assumption, for instance ADHD (McFadden et al., 2005; de Bruin et al., 2006; Stevenson et al., 2007; Martel et al., 2008; Martel, 2009) and autism spectrum disorders (Manning et al., 2001; Milne et al., 2006; de Bruin et al., 2006; De Bruin et al., 2009), both thought to be influenced by prenatal testosterone, are related to 2D:4D as well. One of the most robust findings is the observation that 2D:4D is sexually dimorphic (Hönekopp and Watson, 2010). In general, males have a lower 2D:4D than females, not only in humans, but also in other mammals such as mice (Brown et al., 2002; Manning et al., 2003), rats (Talarovicová et al., 2009), bonobos (McIntyre et al., 2009) and baboons (McFadden and Bracht, 2003; Roney et al., 2004). Despite more evidence needed to validate 2D:4D as an indicator of prenatal testosterone, hundreds of publications in the last decade at least illustrate that 2D:4D is commonly accepted as an indirect biomarker of prenatal testosterone (Voracek, 2014).

Interestingly, 2D:4D has been related to sexually dimorphic behavior, such as aggression (Turanovic et al., 2017), risk taking (Brañas-Garza et al., in press), athletic achievement (Tester and Campbell, 2007), dominance (Manning and Fink, 2008) and according personality traits. Remarkably, surveying the existing literature it seems that the evidence for direct relationships between 2D:4D and personality measures is mixed (effects seem to be difficult to replicate at least). However, some relationships between 2D:4D and behavioral measures that are closely related to the same personality measures seem to be more robust in particular settings. We will conjecture below why these inconsistencies may arise.

Consider the mixed evidence for the relation between risk taking and 2D:4D. Whereas some find a negative relationship between 2D:4D and risk taking measures in both sexes (Dreber and Hoffman, 2007; Garbarino et al., 2011; Chicaiza-Becerra and Garcia-Molina, 2017) others do observe this effect among only men (Brañas-Garza and Rustichini, 2011; Stenstrom et al., 2011) or only women (Hönekopp, 2011) and even a larger amount of published studies did not find any significant association (Apicella et al., 2008; Sapienza et al., 2009; Aycinena et al., 2014; Kim et al., 2014; Drichoutis and Nayga, 2015; Schipper, 2015). Interestingly, some studies provide evidence for the idea that particular characteristics in the environment (Ronay and Von Hippel, 2010) or in the risk taking measure (Brañas-Garza et al., in press) may play a crucial role. Moreover, it is important to be aware that empirical evidence for the relation between 2D:4D and “real-world” risk taking looks much more convincing. For instance, it has been shown that low 2D:4D predicts risky driving behavior in traffic (as measured by the penalty point entries recorded on the driving license; Schwerdtfeger et al., 2010) as well as the likelihood to start a risky finance career (Sapienza et al., 2009). The relation between low 2D:4D and increased profitability of high-frequency financial traders (Coates et al., 2009) has also been explained by an increased tolerance for financial risk (Coates and Page, 2009). 2D:4D seems to be related to criminal risk taking actions too: Some evidence shows that imprisoned criminal offenders have a lower 2D:4D than nonoffenders (Hanoch et al., 2012) and low 2D:4D is related to increased criminal involvement (Ellis and Hoskin, 2015).

If we focus on the relation between 2D:4D and aggression, some recent meta-analyses have shown that the overall effect size of the relationship between 2D:4D and aggression measures is weak (Hönekopp, 2011; Turanovic et al., 2017). However, it is important to take into account that in the majority of the studies adopted in these meta-analyses aggression is measured in artificial settings or by self reports in questionnaires. Again, “real-world” aggressive behavior during sport contests seems to be more consistently related to 2D:4D (Perciavalle et al., 2013; Mailhos et al., 2016). Furthermore, typical studies focus on linear relationships between 2D:4D and aggression without taking the context into account. However, specific characteristics of the context may be crucial to observe any relationship with the dependent measure. At least, some data suggest that cues that point to challenges in the environment (such as aggression or provocation) are essential to observe a relationship between a lower 2D:4D and increased aggression levels (Millet and Dewitte, 2007; Kilduff et al., 2013) or decreased prosociality (Millet and Dewitte, 2009; Ronay and Galinsky, 2011). Accordingly, the relation between unprovoked aggression and 2D:4D in a simulated war game (McIntyre et al., 2007) may have emerged exactly because of the specific context in which the behavior took place.

What may be the reason for these seemingly inconsistent patterns of results? To find an answer, it may be instructive to look at the perspective presented in Millet (2011) and Ryckmans et al. (2015) to understand how specific characteristics of the particular study context and/or dependent measures may be crucial to observe effects between 2D:4D and the variable at hand. Ryckmans et al. (2015) remarked that the effect size of a direct linear relationship between 2D:4D and personality measures of dispositional dominance (see e.g., Manning and Fink, 2008) is weak at best despite more consistent evidence for the negative relationship between 2D:4D and performance in many different sports (Tester and Campbell, 2007; Hönekopp and Schuster, 2010), on the financial markets (Coates et al., 2009) and in cognitive tasks or academic assessments (Brosnan et al., 2011; Hopp et al., 2012; Bosch-Domènech et al., 2014). Moreover, strong relationships between 2D:4D and dominance related behavior or outcomes have been observed in non-human species such as macaques (Nelson et al., 2010) and baboons (Howlett et al., 2012, 2015). Ryckmans et al. (2015) propose that the activation of the dominance system is crucial to observe relations between 2D:4D and dominance and provide experimental evidence showing that male 2D:4D is indeed only associated with a dominant personality trait measure when the dominance system is likely to be activated (that is, after fictitious male-male interaction with another dominant man). This is in line with the perspective of Millet (2011), who argued that 2D:4D would only predict dominant-related behavior in those situations where status is at stake.

This perspective is consistent with empirical findings on circulating testosterone levels. Whereas a growing body of evidence points to the absence of a relationship between 2D:4D and circulating testosterone levels (Muller et al., 2011), low 2D:4D may reflect increased sensitivity to circulating levels of testosterone: Some recent studies show that testosterone administration only influences behavior for men and women with low 2D:4D (Carré et al., 2015; Buskens et al., 2016; Chen et al., 2016). In line with the biosocial model of status, testosterone seems to encourage behavior that is instrumental to dominate others (Mazur and Booth, 1998) and testosterone has especially high predictive validity in those situations when status is at stake (Newman and Josephs, 2009). Therefore, the reasoning that 2D:4D especially predicts dominant-related behavior in status challenging situations is consistent with this account.

In line with the perspective that testosterone is especially predictive when status is at stake we argue that 2D:4D is more likely to be related to status striving in specific, predictable situations than to unspecified measures of general risk taking, dominance, aggression or any other behavior *per se*. Based on this approach we would predict that only when status is at stake relationships between 2D:4D and context-specific goal-directed behavior would emerge (be it risk taking, aggression or even pro-social behavior). Following this reasoning, it is likely that for instance the relation between low 2D:4D and higher levels of aggressive behavior in soccer (Perciavalle et al., 2013; Mailhos et al., 2016) may be driven by the increased chance to win the particular game, but that 2D:4D and general personality measures of aggression are not related when measured in a controlled lab setting. First, status striving motivations are typically not activated when personality measures of aggression are assessed. Second, aggression is only one specific path towards status: whereas it may be functional to attain status in competitive and violent environments aggressive responses may also lead to the opposite effect (or be not effective at all) in other settings. At least some evidence is consistent with this idea as it has been shown that personality measures of aggression are not related to 2D:4D when people are exposed to a non-violent video, but that the relationship between 2D:4D and aggression emerges after exposure to a violent video (Millet and Dewitte, 2007; Kilduff et al., 2013).

Furthermore, the relation between 2D:4D and financial risk taking may predominantly emerge in experimental settings when the behavior is financially incentivized (Brañas-Garza et al., in press) as only higher actual payoffs in the experimental session are able to enhance relative status compared to other participants in the same experimental session. Similarly, relations between 2D:4D and “real-world” risk taking behavior may only arise when the risk one takes may lead to an increased status position. Whereas it has been claimed that increased tolerance for financial risk (Coates and Page, 2009) explains increased profitability of high-frequency financial traders (Coates et al., 2009) we would suggest otherwise: As profitability is status enhancing in this financial context, taking more risk can be considered the only viable option to potentially make the most profits. Thus, the urge to attain status is possibly a more important driving force than the proposed increased risk tolerance (Millet, 2009).

Given our interpretation of inconsistencies in the literature, we set up a study to investigate whether status relevance of the specific context is indeed important in the study of the relation between 2D:4D and any goal-directed (i.e., potentially status-enhancing) behavior. Based on our reasoning we would predict a relationship between any behavior as long as it qualifies as a mean to enhance status in that specific context, but not if status is not relevant in the particular context at hand (and thus the same behavior is not functional anymore to attain status). We decided to focus on decisions without any financial outcome as merely the financial aspect by itself could already change the meaning of the decision. We simply manipulated one aspect of the context so that the same decision is considered functional to attain status or not. Based on our reasoning, we only expect a relationship between 2D:4D and the decision at hand when the decision is functional to attain status. More concretely, we provided a fictitious sports competition scenario in which winning either increased status (an important competition) or was status irrelevant (an unimportant competition). Interestingly, chances to win typical sports competitions can not only be increased by exercise, motivation, aggression, risk taking or physical superiority but also by the use of a wide spectrum of performance enhancing products, going from (legal) supplements to (illegal) doping. Therefore, we asked our participants about their evaluation of different products (both legal and illegal) that could potentially enhance performance. We included both legal and illegal products to create a realistic scenario (both types of products are generally perceived to be common practice in cycling competitions given the anecdotal evidence in popular media that professional cyclists make use of these). As these products are only functional to attain status in the status relevant condition, we expect that a relation between 2D:4D and attitude towards these performance enhancing means only emerges in the status relevant condition. More specifically, we predict that low 2D:4D men will generally be more positive about such performance enhancing means in the status relevant than in the status irrelevant situation. We do not make any *a priori* prediction with regard to the nature of the means (i.e., illegal vs. legal). By adopting this factor it may also provide insights into how far-reaching low 2D:4D men’s ambitions may go. Albeit we make use of an imagination exercise and the attainment of status is therefore purely fictitious (i.e., a construct of participant’s mind) in our experiment. We consider the design a rather conservative test of our hypothesis. If we observe a result that is consistent with this hypothesis despite the “imagination” part and lack of monetary incentivization, then a fortiori we would expect our hypothesis to hold in a framework with real, financially incentivized decisions.

## Materials and Methods

One hundred and nine male students received partial course credits for their participation in the study. This study was carried out in accordance with the recommendations of the ethical guidelines of the faculty of Economics and Business Administration of the Vrije Universiteit Amsterdam with informed consent from all subjects. All subjects gave informed consent in accordance with the Declaration of Helsinki. The protocol was approved by the FEWEB Research Ethics Review Board.

Upon arrival in the laboratory, each participant was assigned to a computer in a partially enclosed carrel. Participants did not see one another and could not talk. A maximum of 14 students participated at the same time. Participants were randomly assigned to one of two between-subjects conditions: a status-relevant vs. status-irrelevant condition. In the status-relevant condition, we asked participants to imagine that they are a professional cyclist and participate in the *most* important cycling race of their season. In the status-irrelevant condition on the other hand, we asked them to imagine to participate in the *least* important cycling race of their season. We chose to change only one word in the introduction to keep everything else constant. The meaning of performance changes depending on the specific context (least vs. most important): The striving to attain status (i.e., winning the race) is only activated in the context of an important race. After this introduction, we asked to what extent (on a 7-point Likert scale; 1: definitely not; 7: definitely yes) they would make use of different means to enhance their performance in the race: nutritional supplements (e.g., a protein shake), prohibited substances (e.g., EPO) and technological fraud (e.g., a hidden engine in the racing bike). Further, we asked them to rate on 7-point Likert scales how bad (=1) vs. good (=7) as well as how unethical (=1) vs. ethical (=7) each of these means are to enhance performance (see for descriptives Table 1). First, we composed a “legal means attitude” vs. “illegal means attitude” by averaging the three items related to nutritional supplements (α = 0.85) and averaging the six items related to the prohibited means (*α* = 0.69).

**Table 1:** Mean (*M*) and Standard Deviation (*SD*) of dependent measures (variable and single items) of the total sample and each condition.

	Total sample (*N* = 109)		Status irrelevant (*N* = 53)		Status relevant (*N* = 56)	
Variable/Measure	*M*	*SD*	*M*	*SD*	*M*	*SD*
**Nutritional Supplements**	**5.75**	**1.46**	**5.37**	**1.68**	**6.10**	**1.12**
Bad/Good	5.82	1.49	5.60	1.61	6.13	1.34
Unethical/Ethical	5.83	1.60	5.51	1.79	6.16	1.34
Intention	5.60	1.86	5.00	2.10	6.16	1.40
**Prohibited Substances**	**1.51**	**0.89**	**1.47**	**0.87**	**1.54**	**0.92**
Bad/Good	1.85	1.62	1.77	1.58	1.93	1.66
Unethical/Ethical	1.32	0.92	1.43	1.20	1.21	0.53
Intention	1.35	0.94	1.21	0.79	1.48	1.04
**Technological Fraud**	**1.44**	**0.70**	**1.38**	**0.64**	**1.51**	**0.75**
Bad/Good	1.67	1.46	1.51	1.31	1.82	1.59
Unethical/Ethical	1.33	0.84	1.40	0.99	1.27	0.67
Intention	1.33	0.84	1.23	0.67	1.43	0.97

*A priori*, we determined to focus on right hand 2D:4D as androgenization is suggested to have a stronger impact on the right than on the left hand (e.g., Williams et al., 2000; McFadden and Shubel, 2002), gender differences are larger for right-hand 2D:4D (Hönekopp and Watson, 2010) and the right hand is more commonly used in previous research (Brañas-Garza and Rustichini, 2011). Hand scans were taken at the end of the session with a high-resolution scanner (Canon Lide 120) and afterwards two independent raters measured (by means of Photoshop CC 2015) the length of index (2nd) and ring (4th) finger. Finger lengths were measured from the bottom crease when there was a band of creases at the base of the digit. Ratios of both raters were highly correlated (*r* = 0.87), speaking towards the accuracy of the measurement. We averaged both ratios to obtain one single measure for 2D:4D and make use of this averaged 2D:4D in our analyses.

## Results

**Figure 1:**
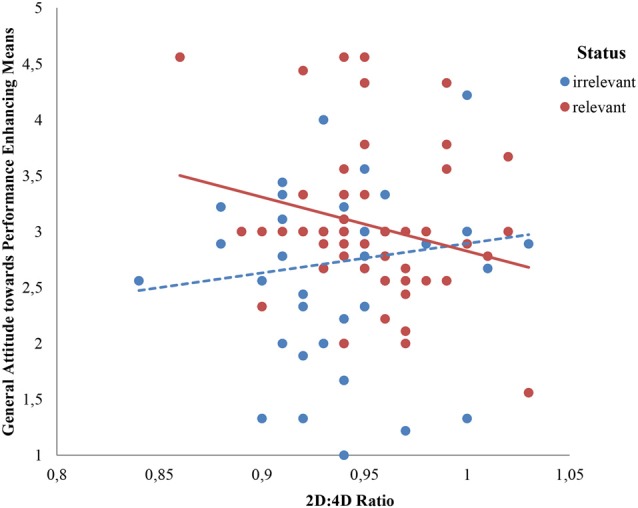
Attitude towards performance enhancing means as a function of 2D:4D ratio and status relevance condition.

We used both attitude measures as dependent variables (within: legal vs. illegal) in a mixed design with 2D:4D (mean-centered) and status relevance (between subjects: status relevant vs. irrelevant) as independent variables. A mixed-design analysis of variance assessed effects of the status relevance manipulation and 2D:4D on the attitude towards legal and illegal means to improve performance, which were included as repeated measures. We observed a more positive attitude towards legal (*M* = 5.75, *SD* = 1.46) than illegal means (*M* = 1.48, *SD* = 0.72, *F*_(1,105)_ = 818.90, *p* = 0.000, partial *η*^2^ = 0.87). Further, a main effect of status relevance on general attitude towards performancing enhancing means emerged (*F*_(1,105)_ = 7.97, *p* = 0.006, partial *η*^2^ = 0.07), which was moderated by the nature of the means (*F*_(1,105)_ = 5.62, *p* = 0.02, partial *η*^2^ = 0.05). Whereas status relevance influenced the attitude towards legal means (*M*_status relevant_ = 6.10, *SD* = 1.12 vs. *M*_status irrelevant_ = 5.37 *SD* = 1.68; *F*_(1,105)_ = 8.56, *p* = 0.004, partial *η*^2^ = 0.08), it did not change attitude towards illegal means (*M*_status relevant_ = 1.52 vs. *M*_status irrelevant_ = 1.42, *p* = 0.53, partial *η*^2^ = 0.004). More interestingly and in line with our predictions, we also observed a marginally significant interaction effect between status relevance and 2D:4D (*F*_(1,105)_ = 3.90, *p* = 0.05, partial *η*^2^ = 0.04). No other effects turned out to be significant (neither within or between; all *p*s > 0.14). To be able to study the interaction between status relevance and 2D:4D in more detail we first calculated a general “attitude towards performance enhancing means” score by averaging the 9 item scores on the three performance enhancing means (*α* = 0.68) and used this measure in the remaining analyses. We aimed to provide insight into this interaction between 2D:4D and status relevance by: (1) calculating Spearman correlation coefficients between 2D:4D and general attitude scores within both conditions to examine in which condition 2D:4D and attitude scores are related; and (2) performing a spotlight analysis (Irwin and McClelland, 2001; Spiller et al., 2013) as such analysis allows us to examine the effect of status relevance at different levels of 2D:4D. This analysis provides insights whether this effect of status relevance is especially driven by low 2D:4D men, high 2D:4D men or both. In accordance with our hypothesis, 2D:4D and the general attitude score were not related in the status irrelevant condition (Spearman’s correlation coefficient *r* = 0.10, *p* = 0.50), but a negative relationship emerged when the situation described was status relevant (Spearman’s correlation coefficient *r* = −0.27 *p* = 0.04; see Figure 1). The results from the spotlight analysis were also consistent with our prediction: For low 2D:4D men (one standard deviation below the mean), the attitude towards performance enhancing means in the status relevant condition was higher than the attitude towards these means in the status irrelevant condition (*M*_status relevant_ = 3.25 vs. *M*_status irrelevant_ = 2.66, *β* = 0.30, *SE* = 0.095, *t*_(105)_ = 3.10, *p* = 0.002). For high 2D:4D men (one standard deviation above the mean), the status relevance of the situation did not influence the attitude towards performance enhancing means (*M*_status relevant_ = 2.91 vs. *M*_status irrelevant_ = 2.85, *β* = 0.03, *SE* = 0.10, *t*_(105)_ = 0.31, *p* = 0.76).

## Discussion

In line with a status drive perspective on 2D:4D, our findings indicate that low 2D:4D men are generally more positive towards performance-enhancing means to win a cycling competition when they believe that the competition at hand is important, but not so when the competition is not important. If low 2D:4D men would take legalness of means into account in their need to achieve status, a three-way interaction should have been observed. However, we did not find any evidence for a differentiation between legal (nutrition supplements) and illegal (EPO, a hidden engine in the bike) means thereby suggesting that low 2D:4D men may be more inclined “to do whatever it takes to win” when stakes are high, but not when the outcome is irrelevant to attain personal status.

If this conclusion is correct, relationships between 2D:4D and any attitude, trait or behavior (be it greedy, impulsive, unethical, altruistic, selfish,…) may emerge as long as these particular attitudes, traits and behaviors help to attain status in that specific situation. However, if the focal behavior is related to an outcome irrelevant to one’s own status position, we do not expect any relationship between 2D:4D and the specific behavior at hand. Some recent evidence in a business context speaks towards this idea: lower use of prohibitive voice (i.e., expressing concerns about practices, behavior, incidents that may be harmful for the organization) is related to a low 2D:4D among low-ranked, but not among high-ranked employees (Bijleveld and Baalbergen, 2017). Albeit speculative, they argue (in line with our reasoning) that prohibitive voice in this particular setting can be considered status relevant for low-ranked, but not for high-ranked employees as it is important for low-ranked employees not to express prohibitive voice to attain or at least maintain status, whereas the use of prohibitive choice does not have any consequence for high-ranked employees (Bijleveld and Baalbergen, 2017).

We believe that a status striving perspective on 2D:4D may shed light on how 2D:4D may drive (economic) decisions. The failure of some studies to find a relationship between 2D:4D and attitudes or behavior may be due to an omitted variable problem, i.e., context: Depending on the particular context, the same behavior or attitudes may be functional in terms of possibilities to increase status or not. Only when considered functional in a specific setting, we would predict a relationship with 2D:4D. For instance, in a recent study it has been shown that 2D:4D only predicts risk taking with real monetary incentives (Brañas-Garza et al., in press). This observation is consistent with our reasoning considering that especially the context with incentivized choices is status-relevant: larger payoffs may directly lead to a higher perceived relative status among the sample of participants in the study. On the other hand, risk attitudes are by itself not directly related to any status-relevant outcome, which may explain why more often no association has been observed between 2D:4D and attitudinal risk taking measures. Our reasoning at least suggests that low 2D:4D men may be especially prone to take (financial) risk when they know that the potential outcome of the risk they take is status-enhancing, even when it is illegal or criminal (consistent with Hanoch et al., 2012; Ellis and Hoskin, 2015).

Following a similar reasoning, monetary incentives may not only change the meaning of financial risk responses but also of other behavioral measures. For instance, Neyse et al. (2016) found that low male 2D:4D is related to higher overconfidence levels when men are asked to predict own performance on a cognitive reflection test (as measured by overestimation, i.e., the individual estimate of the number of correct answers on a cognitive reflection test minus the actual number of correct answers on this test). Still, their effect only held when performance prediction accuracy is not monetarily incentivized: when more accurate predictions are financially rewarded the relationship between 2D:4D and overconfidence actually reverses (Neyse et al., 2016). Following our rationale, we predict that overconfidence will increase or decrease among low 2D:4D men depending on its functionality to attain higher status. Overconfidence has been considered as a way to obtain status (Anderson et al., 2012), and the observed relationship between lower 2D:4D and higher overconfidence levels is thus consistent with our reasoning. However, incentivization of accuracy may actually change the meaning of the measurement. Under the assumption that larger pay-offs in the study at hand may directly lead to a higher perceived relative status among study participants, increased accuracy—and thus lower overconfidence—is actually functional to attain status in this particular setting.

Whereas our perspective may shed light on some inconsistencies in the 2D:4D literature, there is a need to further improve the theoretical perspective to provide insight into other inconsistencies. For instance, when taking a look at the relationship between 2D:4D and prosocial behavior, there have been observed both positive (Buser, 2012), negative (Millet and Dewitte, 2009) and curvilinear (Millet and Dewitte, 2006; Brañas-Garza et al., 2013; Galizzi and Nieboer, 2015) relationships in seemingly neutral situations as well as positive relationships in specific potentially “challenging” situations (Millet and Dewitte, 2009; Ronay and Galinsky, 2011). Whereas both proself and prosocial behavior have been considered as ways to attain status (Millet and Dewitte, 2009), it remains difficult to understand the inconsistency between findings in this stream of literature from the perspective we provide in the current manuscript. Some findings show how contextual characteristics are able to shift the relationship between 2D:4D and choices in ultimatum and dictator games (Van den Bergh and Dewitte, 2006; Millet and Dewitte, 2009; Ronay and Galinsky, 2011) and incentivization has been considered to be important as well (Brañas-Garza et al., 2013). Therefore, it seems to be crucial to consider the context in which the behavior took place as well as the specific nature of the measurement (e.g., incentivized or not, type of economic game, etc.). Though, the overall pattern of results in this domain remain difficult to explain from our perspective. For instance, our perspective does not allow to make any inference on how people with a “medium” 2D:4D may react differently compared to low and high 2D:4D people (Brañas-Garza et al., 2013; Galizzi and Nieboer, 2015). Still, we believe that the relations between 2D:4D and proself/prosocial choices are at least influenced by the perceived functionality to attain status in the specific context in which the study took place albeit other aspects seem to be crucial as well.

At least, our pattern of results corroborates the viewpoint that male 2D:4D is negatively related to performance in many domains because of the need for status. We suggest in line with Millet (2009) and Millet and Dewitte (2008) that low 2D:4D men may also self-select into those domains in which they excel (be it sports, music, cognitive performance or even performance on financial markets) as long as their superiority in that specific domain provides them with a feeling of higher relative standing. Remarkably, this self-selection perspective would predict a relationship between 2D:4D and level of competition (e.g., lower 2D:4D in national vs. professional and/or recreational teams; Frick et al., 2017; Manning and Taylor, 2001), but not necessarily within competition. For instance, consider low 2D:4D men without the necessary skills to be part of a professional soccer team but still remain playing soccer at low level. Given the absence of superior performance they probably do so because of intrinsic motivation (i.e., the pleasure of the game) and not for the sake of status.

Our context-dependent perspective can be considered in line with the recent hypothesis that many of the relations between low 2D:4D and improved performance in sports (as well as in other domains) may be driven by the association between low 2D:4D and pronounced spikes of testosterone in challenge situations (Manning et al., 2014). Therefore one avenue for further research could focus on the interplay between circulating testosterone and 2D:4D by: (a) measuring circulating testosterone in different settings and investigate whether the relationship between 2D:4D and status-driven behavior is induced by enhanced circulating testosterone levels in these settings and thus increased testosterone sensitivity of low 2D:4D individuals; or (b) testing whether low 2D:4D predicts the production of testosterone levels in challenge situations. Such studies could at least provide further insight into the biological basis for the presumed relation between 2D:4D and status striving. We also would like to point out that our imbalanced sex ratio in the lab (only men participated) may have induced a more competitive setting by itself (see Griskevicius et al., 2012) and thereby increased circulating testosterone levels in general. Still, this assumption remains open for future research as well as the plausible hypothesis that a male biased sex ratio may have led to our specific pattern of results.

Finally, it is important to realize as well that it remains difficult to *ex ante* identify those contexts in which a particular behavior is considered functional to attain status or not. Whereas we are able to integrate many inconsistent findings in the literature based on this status striving perspective, further elaboration of the theoretical perspective is needed to get a better understanding of under what specific circumstances we may expect relationships between 2D:4D and other variables of interest. Therefore, another interesting avenue for further reseach is the study of the relation between 2D:4D and performance indices or specific decisions that may be considered functional or not to attain status in different contexts to provide insights into the generalizability of our findings. Further validation of our hypothesis would be especially desirable in incentivized laboratory or field studies in which (real) decisions need to be taken that are either functional or not to attain status in that particular context.

To conclude, in the present article we presented a theoretical perspective that provides an interpretation of inconsistencies in current 2D:4D literature. Further, we provided some empirical evidence for our reasoning that low 2D:4D men may do whatever it takes to attain status, thereby stressing the functionality of specific behavior towards this status goal in the particular context at hand. We hope that our interpretations, propositions and discussion are helpful in the formation and/or further development of a highly needed theoretical perspective to understand how 2D:4D influences behavior and that the present analysis is at least helpful to identify interesting paths for future research.

## Author Contributions

KM designed the study. FB carried out the experiment and collected data. KM and FB analyzed the data. KM wrote the manuscript with support from FB. Both authors agree to be accountable for the content of the work.

## Conflict of Interest Statement

The authors declare that the research was conducted in the absence of any commercial or financial relationships that could be construed as a potential conflict of interest.
